# Identification of Novel Non-Nucleoside Inhibitors of Zika Virus NS5 Protein Targeting MTase Activity

**DOI:** 10.3390/ijms25042437

**Published:** 2024-02-19

**Authors:** Diego Fiorucci, Micaela Meaccini, Giulio Poli, Maria Alfreda Stincarelli, Chiara Vagaggini, Simone Giannecchini, Priscila Sutto-Ortiz, Bruno Canard, Etienne Decroly, Elena Dreassi, Annalaura Brai, Maurizio Botta

**Affiliations:** 1 Department of Biotechnology, Chemistry and Pharmacy, University of Siena, via Aldo Moro 2, 53100 Siena, Italy; 2 Department of Experimental and Clinical Medicine, University of Florence, Viale Morgagni 48, 50134 Florence, Italy; mariaalfreda.stincarelli@unifi.it (M.A.S.); simone.giannecchini@unifi.it (S.G.); 3 AFMB, Aix-Marseille University, CNRS, UMR 7257, Case 925, 163 Avenue de Luminy, Cedex 09, 13288 Marseille, France; priscila.sutto-ortiz@univ-amu.fr (P.S.-O.);

**Keywords:** NS5, non-nucleoside inhibitors, ZIKV, DENV, antiviral agents, LD50

## Abstract

Zika virus (ZIKV) is a positive-sense single-stranded virus member of the *Flaviviridae* family. Among other arboviruses, ZIKV can cause neurological disorders such as Guillain Barré syndrome, and it can have congenital neurological manifestations and affect fertility. ZIKV nonstructural protein 5 (NS5) is essential for viral replication and limiting host immune detection. Herein, we performed virtual screening to identify novel small-molecule inhibitors of the ZIKV NS5 methyltransferase (MTase) domain. Compounds were tested against the MTases of both ZIKV and DENV, demonstrating good inhibitory activities against ZIKV MTase. Extensive molecular dynamic studies conducted on the series led us to identify other derivatives with improved activity against the MTase and limiting ZIKV infection with an increased selectivity index. Preliminary pharmacokinetic parameters have been determined, revealing excellent stability over time. Preliminary in vivo toxicity studies demonstrated that the hit compound **17** is well tolerated after acute administration. Our results provide the basis for further optimization studies on novel non-nucleoside MTase inhibitors.

## 1. Introduction

Viruses represent an important challenge of our century. Though 219 types of viruses can infect humans [1], this number represents just the tip of the iceberg, with millions of species that affect plants and animals and can potentially affect humans as well. From 2015 to 2016, a Zika fever epidemic caused by the Zika virus (ZIKV) spread and affected people from Brazil and South America, with thousands of babies born with brain damage. Although the incidence of ZIKV infection has declined substantially since 2016, ZIKV transmission persists to the present [2]. Starting from the 2016 epidemic, large efforts have been dedicated to the search for effective treatments; nevertheless, no drugs nor vaccines are currently available in clinics and Zika syndrome is still included in the list of rare orphan diseases [3]. Several compounds including nucleoside analogs that target viral polymerase, protease inhibitors, or antivirals with broad-spectrum application against HCV and flaviviruses like sofosbuvir [4,5] have been investigated in animal efficacy models or in clinical trials. In this context, the drug-repurposing approach has contributed to the identification of compounds effective in in vitro and preclinical studies. For example, the antimalarial drug chloroquine has been shown to limit viral entry, with in vitro EC_50_ values of about 5 µM, but it is barely active in animal models [6,7]. Among the nucleoside analogs, the broad-spectrum antiviral ribavirin is only moderately active against ZIKV, with EC_50_ values in the high micromolar range [8]. Inhibitors targeting the helicases NS3 and the bifunctional NS5 protein, such as suramin, sofosbuvir, and sinefungin, also appear to be promising in in vitro assays [9,10]. However, further studies are needed to confirm their efficacy in animal models. Considering that novel epidemics can be just around the corner, it is important to find specific treatments to block this viral infection.

Nonstructural protein 5 (NS5) is an attractive antiviral target. NS5 contains a C-terminal domain that contains the RNA-dependent RNA polymerase activity essential for RNA replication/transcription and an N-terminal methyltransferase (MTase) domain [11]. The MTase catalyzes two different methylation reactions, targeting the N7-guanosine position of the guanosine moiety of the cap structure and the (2′-O) of the ribose of the first nucleotide (N1) of viral RNA [12], respectively.

The N7 methylation of the cap structure is needed for the recognition of mRNA by Eukaryotic translation Initiation Factor 4E (EIF4E), allowing the initiation of RNA translation into viral proteins [5,13,14]. However, the 2′-O-methylation of the cap structure is a self-marker limiting the RNA recognition by the Retinoic acid-Inducible Gene (RIG-like) receptor RIG-I, which mediates the induction of type I Interferons [15,16]. In addition, this 2′-O-methylation also limits the sequestration of viral RNA by the Interferon-induced protein with tetratricopeptide repeats (IFIT) [17]. Thus, the inhibition of NS5 MTase activity is supposed to impair the translation of viral RNA into proteins and increase the antiviral response at the infection site linked to the Interferon response. This was further established by mutagenesis studies conducted on West Nile virus (WNV) and Dengue virus (DENV) MTases, demonstrating that mutations abolishing methylation reactions are lethal for viral replication [11,18]. Thus, the NS5 MTase is now considered an interesting antiviral target.

The MTase domain of NS5 is composed of two main subpockets: the unique S-Adenosyl-L-Methionine (SAM)-binding pocket and an RNA-binding groove accommodating the RNA substrate in different positions for either N7 or 2′-O methylation. Additionally, given that ZIKV infection can be associated with other arboviruses like DENV [19,20] or Chikungunya (CHIKV) [21], the identification of small-molecule inhibitors targeting the SAM domain may prove useful in treating co-infections.

Accordingly, huge efforts have been made to identify novel small-molecule inhibitors of NS5 using in silico methods. In 2017, Ramharack and Soliman identified a novel potential MTase inhibitor on the commercial ZINC database, the tetrazole ZINC64717952 [22]. Santos et al., through a virtual screening procedure, identified a rhodanine compound, ZINC1652386, as a potential inhibitor of the NS5 MTase domain [23]. Recently, Bharadwaj et al. screened the Life Chemical database and identified different sulfonamides as potential inhibitors of the NS5 MTase of ZIKV [24]. Even if the reported results were promising, none of these compounds demonstrated activity against ZIKV-infected cells, making the search for novel compounds important.

With the purpose of identifying novel inhibitors of the ZIKV NS5 MTase domain with proven activity in antiviral assays and suitable in vitro properties, we applied a multidisciplinary approach that combined in silico methods with biological evaluation and the analysis of in vitro stability and toxicity in vivo. We performed virtual screening by combining molecular docking and molecular dynamics to identify compounds targeting the SAM binding site, which is highly conserved among flavivirus MTases. We anticipate that such compounds will exhibit pan-flavivirus antiviral activity.

## 2. Results and Discussion

### 2.1. In Silico Studies

The crystal structure of ZIKV NS5 MTase in complex with the SAM cofactor has been previously resolved by Coutard et al. [11].

**Figure 1:**
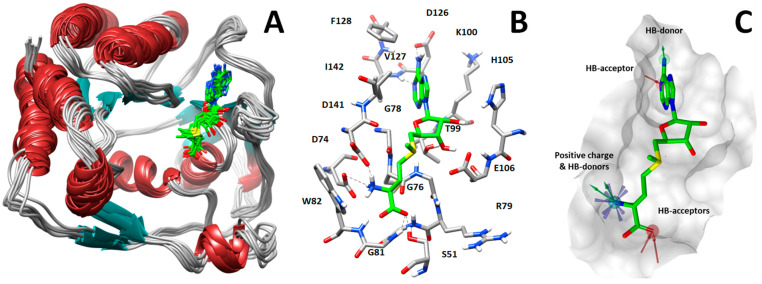
(**A**) Analysis of protein dynamics and ligand–protein interactions. (**B**) Key interactions between SAM (green sticks) and NS5 MTase. (**C**) Pharmacophore model used to perform virtual screening of compounds into the SAM-binding pocket.

Starting from the X-ray structure, we performed molecular dynamics (MD) simulations aimed at analyzing the ligand–protein interactions formed between MTase and SAM (Figure 1A). Based on this analysis, we identified three groups of key SAM–MTase interactions involving the adenine moiety of SAM as well as the amino and carboxylic groups of its methionine portion. In detail, the SAM adenine moiety is localized in a hydrophobic pocket constituted by the lateral chains of Val127, Phe128, and Ile142 and makes hydrogen bonds with the carboxyl oxygen of Asp126 and NH of Val127 backbone, whereas the methionine moiety establishes hydrogen bonds with Ser51, Trp82, and Asp141 sidechains (Figure 1B).

**Figure 2:**
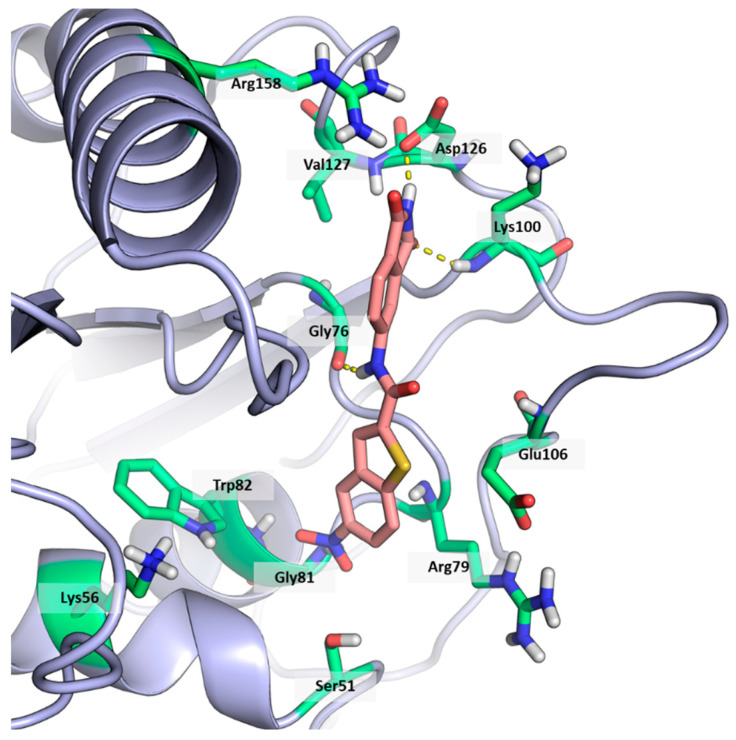
Binding mode of ligand **1**. The hydrogen bonds are represented by yellow dotted lines.

Starting from this result, a virtual screening protocol was built and used to screen a database of about 5 million commercial compounds (see Section 3 for details). Firstly, a pharmacophoric model was developed taking into consideration the most populated interactions (Figure 1C). The pharmacophore was used to perform a first screening, choosing molecules matching the pharmacophore features representing the interactions of the SAM adenine group and at least one of the two groups of features representing the interaction of SAM methionine moieties, with the aim of identifying compounds able to form interactions within both the adenine and the methionine subpockets of the SAM-binding site. The obtained results were then subjected to docking analysis using Glide XP, Glide SP, and GOLD (using the GoldScore fitness function). At the end of each docking procedure, a post-docking filter was applied to check whether the desired pharmacophoric interactions were maintained in the predicted binding modes of the docked compounds. Only the ligands for which a common binding mode matching the desired pharmacophore features was predicted by all the docking software were retained and then subjected to clustering and visual inspection. As a result of the whole workflow, ten compounds were selected, purchased, and subjected to MTase inhibition assays. Among them, ligand **1** (Figure 2, Table 1) revealed an interesting dual-inhibitory activity against ZIKV and DENV MTase, with IC_50_ values of 53 and 69 µM, respectively (Figure S2 in Supplementary Materials).

Starting from this result, extensive molecular dynamics studies were performed to better evaluate the binding mode and the main temporary interactions of **1** within the SAM-binding pocket. Seven replicas of 100 ns were performed with the software Amber16 [25]. The trajectories were analyzed with MM-PB/GBSA [26] to determine the stability of protein–ligand complexes. The extension H-Bonds in the software VMD (Version 1.9.3) [27] was used to determine the occupancy of each hydrogen bond during the simulations.

As shown in Figure 2, the extensive MD simulation study highlighted a pattern of ligand–protein interactions that slightly differed from that initially predicted by docking (Figure S1). The phthalimide headgroup was involved in a bidentate interaction with Asp126 carboxylic acid and Lys100 backbone NH. The amide core group formed an H-Bond interaction with Gly76, and the nitro group in the tail of the compound interacted with Ser51 and Gly81. However, the latter interactions observed during the molecular dynamic simulations, located in the lower portion of the SAM-binding site, were found to be only transiently formed. Therefore, this pocket could be better targeted in an attempt to increase the stability of the protein–ligand complex.

**Figure 3:**
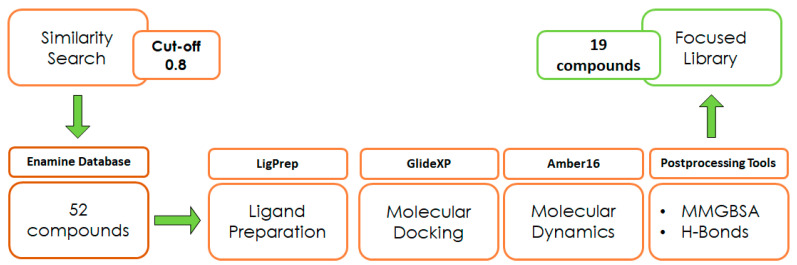
Design of experiment scheme.

With the aim of testing the reliability of the main and transient interactions predicted for ligand 1, we performed a similarity search on the Enamine commercial database to identify suitable analogs of the compound to obtain some SAR data. The similarity search was run on the Enamine real database (around 5 M compounds), applying a cut-off of 0.8 in terms of 2D Tanimoto similarity (Figure 3). The initial pool of selected ligands was processed using LigPrep [28] and docked into the binding site of ZIKV MTase with GlideXP [29,30] using the frame representative of the most abundant cluster of MD. The predicted ligand–protein complexes were then subjected to 100 µs simulation time MD simulations using Amber16 [25]. Molecular proprieties of logP and logS were predicted through the software SwissADME [31] (Table 1). The analysis of the protein–ligand complexes, performed with the molecular mechanics-generalized Born surface area (MM-GBSA) method, was used for the prioritization of compounds (Table S1, Supplementary Materials). As a result, a set of 19 analogs was selected and purchased.

### 2.2. Enzymatic Assays

Selected compounds were evaluated in enzymatic inhibition assays against both ZIKV and DENV 2′-O-MTase activity (For details, see Figure S3 in Supplementary Materials). The screening of compounds at a concentration of 50 µM highlighted a higher inhibitory activity against ZIKV MTase. We thus focused our efforts on ZIKV and determined the IC_50_ values against ZIKV MTase.

As shown in Table 1, the molecules selected from the similarity search were characterized by structural punctiform modifications with respect to the parent compound **1**.

Compound **2**, characterized by the replacement of the phthalimide with an isoindolin-1-one ring, has an IC_50_ of 20 µM, 2.5 times better than the precursor. The alkylation of the phthalimide nitrogen with a methyl improved the activity of compound **3**, with an IC_50_ value of 16.55 µM. Our in silico model suggested that this result is due to the Van Der Waals interactions between the methyl group and the sidechains of Val127 and Phe128; in addition, the methylation of the phthalimide ring reduced the distance between the nitro group and Gly81, creating an additional hydrogen bond. The replacement of phthalimide with a benzene-3-sulfonamide, benzamide, or benzoic acid led to compounds with comparable inhibitory activity (**4**–**6**). Finally, the phthalimide was replaced with a phenyl, obtaining a comparable activity, even in this case (**7**). The replacement of benzothiazole with a benzofuran ring slightly decreased the potency of compound **8**. The results are in agreement with our in silico analysis, which highlights the lower stability of the complex in terms of ΔG values and occupancy values.

Then, we checked the importance of the nitro group by analyzing compounds **9**–**20**. The in silico analysis of the transient interaction suggested that nitro groups are important for the stability of the protein–ligand complex. As reported in Table 1, the absence of a nitro group was detrimental to compound **9**, which was completely inactive. On the contrary, **10** retained an inhibitory potency comparable with **5**. The methylation of amide in meta or in para position completely abolished the activity in compounds **11** and **12**. The absence of nitro groups in compounds **13** and **14** increased the IC_50_ values by about two times. The analysis of the protein–ligand complexes, performed with the MM-GBSA method, highlights higher stability values for compounds characterized by the presence of nitro groups with respect to their corresponding derivatives without the nitro group (i.e., ΔG= −36 and −26 kcal/mol for **3** and **14**, respectively). Similar results were obtained with compounds **2** and **13** with ΔG values of −34 and −29 kcal/mol, respectively. The replacement of nitro with a chlorine or the introduction of a chlorine in the −3 or −7 position retained or slightly decreased the IC_50_ values in compounds **15**–**17**; in contrast, the introduction of a chlorine in the 4-position was detrimental to the activity of **18**. The replacement with methyl or the introduction of methyl in the 7-position led to similar results.

### 2.3. Antiviral Assays

Compounds characterized by the best anti-enzymatic activity were tested against ZIKV in cellular assays. VERO E6 cells were infected with ZIKV at a multiplicity of infection (M.O.I.) of 0.1, and their activities were evaluated by a plaque reduction assay three days post-infection. Ribavirin was used as a reference compound. As reported in Table 2, compound **1**, identified in the first screening, was unable to inhibit viral replication up to a 100 μM concentration. Among compounds identified by the similarity search approach, compound **3** had an inhibitory activity of 28.6 µM, while the best results were obtained with **8** and chlorinated compounds **15**–**17** with IC_50_ values between 0.5 and 2.6 μM. Compound **20**, characterized by the presence of a methyl in the 7-position of the benzothiophene ring, had an IC_50_ of 6.6 μM. The cytotoxicity of the series was determined on the same cell line. Interestingly, all compounds were not toxic at concentrations up to 100 μM. Their selectivity indexes were calculated as the ratio between CC_50_ and IC_50_ values; the results highlight their promising safety profile.

### 2.4. Preliminary Stability Analysis

Phthalimide is a common moiety of many drugs with antifungal or anticancer activity [32]. The lipophilic nature of phthalimide influences its ability to reach target organs in vivo; however, its stability can be influenced by substituents. In vitro stability was thus assessed for the most promising compounds of the series (**8**, **15**, **17**, **20**) to preliminarily identify a lead candidate for further in vivo tests.

The metabolic stability was investigated by incubating selected compounds with human liver microsomes (HLMs) for 1 h at 37 °C. The percentage of non-metabolized compounds and metabolites was analyzed by HPLC-UV-MS. As shown in Table 3, the values range from excellent to good, being higher than 96%. HPLC analysis confirmed the presence of the oxidated metabolites in small percentages.

Finally, compounds were incubated in human plasma at 37 °C for 24 h. All derivatives except **8**, characterized by the presence of the benzofuran ring, had excellent plasma stability, with their half-life being higher than 24 h. Taken together, stability studies confirmed that the series is suitable for further development.

### 2.5. In Vivo Toxicity

**Figure 4:**
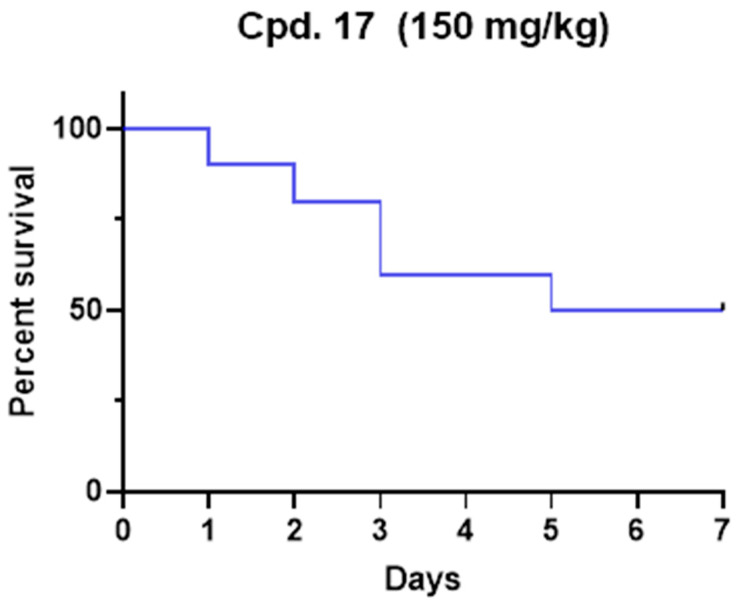
Kaplan–Meier survival rate curve for compound **17** after a single dose administration at the dose of 150 mg/kg.

The median lethal dose (LD_50_) was determined for compound **17** in a *Tenebrio molitor* coleoptera (TMC) toxicity model published previously [33]. Due to ethical reasons, and according to the 3Rs principle, the insect model was preferred to the murine model to preliminarily evaluate the potential in vivo toxicity. After the range-finding study, one microliter of the compound was administered to five TMCs at a single dose of 200 mg/kg, and vitality was observed for seven days. The experiment was repeated until LD_50_ was determined and finally confirmed with ten TMCs. The LD_50_ for **17** was 150 mg/kg of body weight (Figure 4). Our toxicity experiment demonstrates that **17** is less toxic than commonly used drugs such as paracetamol and carbamazepine [33]. Even if complete pharmacokinetics should be performed to determine plasmatic levels, the administered concentration was higher than IC_50_. Considering that no antiviral efficacy models have been developed in insects, future studies with optimized compounds should be translated into suitable models (i.e., mice). Nevertheless, this preliminary result represents the proof of concept that **17** possesses a promising safety profile.

## 3. Materials and Methods

### 3.1. Pharmacophore Modeling and Screening

The pharmacophore model was generated using the software Ligandscout, [34] using the crystal structure of ZIKV NS5 MTase in complex with the SAM cofactor (PDBid 5M5B), and was based on the most stable cofactor–protein interactions observed during the MD simulations performed on the complex. The model includes a total of 7 features: 3 H-bond acceptors, 3 H-bond donors, and 1 positive ionizable feature. Moreover, the excluded volume spheres, representing the region of space that cannot be occupied by the ligands during the screening, were added to the model. A database of about 5 million commercial compounds was used as the screening database. The database was obtained starting from the Aldrich Market Select compound collection, including compounds belonging to many different chemical vendors, which was preliminary pre-filtered to eliminate unsuitable compounds (e.g., compounds with less than 15 heavy atoms, compounds with MW > 700, organometallic compounds). The database was screened using the previously created pharmacophore model. Only compounds matching the pharmacophore features representing the interactions of the SAM adenine group and at least one of the two groups of features representing the interactions of the SAM methionine moiety were retrieved.

### 3.2. Docking Calculation

The crystal structure of the ZIKV MTase, downloaded from the Protein Data Bank (PDBid 5M5B), was prepared using the protein preparation wizard in Maestro suite. The ligands were prepared using LigPrep Software, keeping the main tautomer and protonation state (at the physiological pH value of 7.4). Docking calculation was run using Glide SP, Glide XP, and GOLD (using the GoldScore fitness function) considering the bound ligand of the reference X-ray structure as the center of the docking grid. The filtering of the docking results was performed by superimposing the docked compounds to the pharmacophore model directly from the supplied poses, without changing their coordinates. Only compounds matching the pharmacophore features representing the interactions of the SAM adenine group and at least one of the two groups of features representing the interactions of the SAM methionine moiety were retained.

The compounds selected through the whole consensus docking procedure were clustered based on their 2D Tanimoto similarity, thus forming groups of ligands with Tanimoto scores > 0.7, and one representative of each cluster of ligands was selected for biological evaluation.

### 3.3. Molecular Dynamics Simulation

MD simulation was run using Amber16 software and the input files were generated through the AmberTools17 package. Ligands were parameterized using Antechamber with gaff Forcefied. The MD simulation protocol started with a solvent minimization followed by a system minimization both using the 1500-step steepest descent gradient followed by the 500 conjugate gradient method. The system was heated for 25,000 steps using a Langevin Thermostat from 0 to 100 K followed by constant pressure heating in periodic boundary conditions up to 300 K with a Berendsen barostat, then 1.5 ns equilibration was performed at 300 K with a Langevin thermostat and a Berendsen barostat followed by replicas of 100 ns production each.

The trajectories were evaluated through clustering of the frames on the ligand RMSD, and evaluation of % occupancy of H bonds formed by the ligand during the replicas was performed using the VMD tool. Interactions with occupancy values lower than 10% were considered transient. The MMGBSA calculation was run on the whole trajectories with an offset of 10 frames to reduce calculation time. The salt concentration was set at 0.150 M to mimic the physiological condition. The radius of the probe was set to 1.4 and the Generalized Born setting, igb, was set to 5, to optimize the calculation of the electrostatic part of the solvation-free energy.

### 3.4. Radioactive Enzymatic Assays

Expression and purification of ZIKV NS5 MTase: The coding sequence of the ZIKV NS5 MTase domain (strain H/PF/2013, GenBank accession no. KJ776791.2, aa 4–278) was synthesized by Genscript and cloned into a pQE30 vector (Qiagen, Hilden, Germany) with an N-terminal His6 tag. The protein was expressed and purified following the procedures described in Coutard et al. [12].

Briefly, after expression in Escherichia coli T7 Express Iq (New England BioLabs) in Terrific Broth at 17 °C overnight, bacterial pellets were resuspended in lysis buffer (50 mM Tris-HCl [pH 8], 300 mM NaCl, 5% glycerol, 0.1% Triton, 10 μg/mL DNase I, 2 tablets of EDTA-free antiprotease cocktail [Roche] and 0.25 mg/mL lysozyme). Following cell lysis and clarification, the protein was purified by affinity on immobilized metal affinity chromatography (IMAC, His prep column [GE Healthcare]), followed by size exclusion chromatography (Superdex S75 HR 16/20 column [GE Healthcare]). The NS5-MTase was concentrated and stored at −20 °C after adding glycerol to a final concentration of 40%.

Filter-binding assay: The transfer of tritiated methyl from ^[3H]^SAM onto RNA substrate was monitored by a filter-binding assay (FBA), performed according to the method described previously [35]. FBA was carried out in a reaction mixture [40 mM Tris-HCl (pH 8.0), 1 mM DTT, 2 μM SAM, and 0.1 μM 3H-SAM (Perkin Elmer, Shelton, CT, USA)] in the presence of 0.7 μM synthetic ^7m^GpppAC_4_ and ZIKV NS5 MTase (500 nM). The enzyme was first mixed with an increasing concentration of inhibitors, previously suspended in dimethyl sulfoxide (DMSO), before the addition of RNA substrate and SAM, and then incubated at 30 °C. The final DMSO concentration in reactions was 5%. Reaction mixtures were stopped after 30 min incubation by their 10-fold dilution in ice-cold water. Samples were transferred to diethylaminoethyl (DEAE) filtermat (Perkin Elmer, Shelton, CT, USA) using a Filtermat Harvester (Packard Instruments, Meriden, CT, USA). The RNA-retaining mats were washed twice with 10 mM ammonium formate pH 8.0, twice with water, and once with ethanol before drying. They were soaked with scintillation fluid (Perkin Elmer, Shelton, CT, USA), and 3H-methyl transfer to the RNA substrates was determined using a Wallac MicroBeta TriLux Liquid Scintillation Counter (Perkin Elmer, Shelton, CT, USA). For IC_50_ measurements, values were normalized and fitted with Prism (GraphPad software version 10.1.1) using the following equation: Y = 100/(1 + ((X/IC50) ^ Hillslope)).

### 3.5. ZIKV Inhibitory Viral Plaque Reduction Assay

Vero E6 cells were used for the inhibitory viral plaque reduction assay. The cell propagation medium was Dulbecco’s Modified Eagle’s Medium (DMEM; SIGMA, Milano, Italy) supplemented with 10% Fetal Bovine Serum (FBS; SIGMA) and 1% Penicillin/Streptomycin (SIGMA). ZIKV viral stock, consisting of cell-free supernatants of acutely infected Vero E6 cells, was aliquoted and stored at −80 °C until use. Titration of the viral stocks as plaque-forming units (PFUs) was carried out in Vero E6 cells. Zika was used to infect Vero E6 cells in duplicate and viral plaques were visualized 4 days following infection. Briefly, 6-well plates were seeded with 2.5 × 10^5^ cells in 3 mL of growth medium and kept overnight at 37 °C with 5% CO_2_. On the day of infection, after removal of the growth medium, cell monolayers at 80–90% confluence were infected with Zika viral stock with a multiplicity of infection (MOI) of 0.1 in a final volume of 0.3 mL and incubated for 1 h at 37 °C with 5% CO_2_. Then, cells were washed with PBS 1X, and 30 μL dimethylsulfoxide (DMSO) alone (viral positive control) or with 10-fold serial dilutions of anti-NS5 inhibitory compounds were immediately added in duplicates together with 300 μL of fresh DMEM complete medium (compound final concentrations of 100, 10, 1, 0.1, and 0.01 μM). Ribavirin (1-β-d-Ribofuranosyl-1,2,4-Triazole-3-Carboxamide; SIGMA) diluted in DMSO was used as an inhibitory reference control. Then, the overlay medium composed of 0.5% Sea Plaque Agarose (Lonza, Basel, Switzerland) diluted in propagation medium was added to each well. After 4 days of incubation at 37 °C, the monolayers were fixed with methanol (Carlo Erba Chemicals, Milan, Italy) and stained with 0.1% crystal violet (Carlo Erba Chemicals), and the viral titers were calculated by plaque-forming unit (PFU) counting. The percentage of plaque reduction activity was calculated by dividing the average PFU of treated samples by the average of DMSO-treated samples (viral positive control). Fifty percent inhibitory concentrations (IC_50_) were calculated using the predicted exponential growth function in Microsoft Excel. Mean IC_50_ + standard deviations (SDs) were calculated using all replicates. All experiments were repeated at least twice. All experimental procedures were conducted under biosafety level 3 containment.

### 3.6. Cytotoxicity Assay

Monolayers of 2.5 × 10^4^ Vero E6 cells per well were grown in flat-bottom 96-well culture plates and allowed to adhere overnight. Then, when the cell layers were confluent, the medium was removed, the wells were washed twice with PBS, treated with 100 µL of DMEM with 10 µL DMSO alone (cell positive control) or with various concentrations of anti-NS5 inhibitory compounds under study (compound final concentrations of 100, 10, 1, 0.1, and 0.01 μM), and incubated for 3 days at 37 °C in a CO_2_. After treatment, an MTT (3-(4,5-dimethylthiazol-2-yl)-2,5-diphenyltetrazolium bromide) kit (Roche, Milan, Italy) was used according to the supplier’s instructions, and the absorbance of each well was determined using a microplate spectrophotometer at a wavelength of 570 nm. Cytotoxicity was calculated by dividing the average optical density of treated samples by the average of DMSO-treated samples (cell positive control).

### 3.7. UV/LC-MS Methods

For the ADME property determination, an Agilent 1260 Infinity HPLC-DAD interfaced with an Agilent MSD 6130 (Agilent Technologies, Palo Alto, CA, USA) was used. Chromatographic conditions were optimized using a Phenomenex Kinetex C18-100 Å column (150 × 4.6 mm) with 5 µm particle size and gradient elution with a binary solution (eluent A: H_2_O; eluent B: ACN; both eluents were acidified with formic acid 0.1% *v*/*v*) at room temperature. The LC analyses started with 5% of B (from t = 0 to t = 1 min), then B was increased to 95% (from t = 1 to t = 10 min) and then kept at 95% (from t = 10 to t = 19 min) and finally returned to initial conditions in 1.0 min. The flow rate was 0.6 mL/min and the injection volume was 10 µL.

### 3.8. Metabolic Stability in HLMs (Human Liver Microsomes)

The test was performed as previously reported with minor modifications [36]. Each compound was dissolved in DMSO and phosphate buffer (0.025 M, pH 7.4), and human liver microsomes (0.2 mg/mL) and a NADPH-regenerating system in MgCl_2_ 48 mM were added to a final volume of 500 µL. This solution was incubated for 1 h at 37 °C and then the reaction was stopped and quenched using 1 mL of ACN. The reaction mixtures were then centrifuged (4000 rpm for 10 min) and the supernatant was taken, dried under nitrogen flow, and then resuspended in 100 µL of methanol. The parent drug and metabolites were subsequently determined using the chromatographic conditions described above. The percentage of non-metabolized compounds was calculated by comparison with reference solutions. The determination of metabolic stability for each compound was performed in three independent experiments.

### 3.9. Stability Test in Human Plasma

A stock solution of the target compound in DMSO (2 mM) was incubated in a test tube with pooled human plasma (55.7 mg protein/mL) and HEPES buffer (25 mM, 140 mM NaCl pH 7.4) up to a final volume of 1.0 mL. The mixture was incubated at 37 °C, and at set time points (0.0, 0.5, 1, 4, 8, 24 h), samples of 50 µL were taken, mixed with 400 µL of ACN to stop the reaction of plasmatic esterases, and centrifuged at 5000 rpm for 15 min. The supernatant was removed and analyzed using the LC-UV/MS method previously described. The half-life (t_½_) was calculated according to the following equation:

$$t½=\frac{ln\;2}{k}$$

where k represents the substrate elimination constant. For each compound, the determination was performed in three independent experiments.

### 3.10. Animals

*Tenebrio molitor* coleoptera (TMC) and pupae for acute toxicity tests were fed at the University of Siena (Siena, Italy) on a diet previously published. Pupae and adult TMC were reared in semi-dark conditions at a temperature of 27 ± 1 °C and a relative humidity (RH) of 40–50%.

### 3.11. Median Lethal Dose Assessment

Median lethal dose assessment was carried out as previously published [28]. Three days after the mutation, TMCs were randomly chosen. The appropriate amount of compound **17** was solubilized at the appropriate dose expressed in mg/kg of insect weight. Using a Hamilton syringe (7001 KH, volume 1 µL, needle size 25 s, cone tip), 1 µL of the solution was administered directly into the hemocoel between the pronotum and elytron. The dose-finding investigation was carried out starting from a dose of 200 mg/kg. Five TMCs were given the chosen dose, and survival was tracked for seven days to determine the median lethal dose (LD_50_). The protocol was repeated until the LD_50_ was established, and the results were confirmed in ten TMCs.

## 4. Conclusions

The growing number of viral outbreaks in recent years highlights the urgent need to provide effective antivirals. In this context, host-targeted and virus-targeted compounds are both needed as complementary approaches.

Herein, we focus our efforts on the analysis of the ZIKV NS5 MTase enzyme. Molecular dynamics simulations were carried out to analyze the interactions between MTase and SAM. The analysis allowed us to identify three groups of important SAM-MTase interactions involving the amino and carboxylic groups of SAM’s methionine, as well as the adenine moiety of SAM. A pharmacophore model was built to screen commercial databases. As a result, we identified a novel class of phthalimide small-molecule inhibitors of ZIKV MTase.

The computational study allowed us to preliminarily define the binding mode of compound **1**. Through iterative cycles of screening, the other 19 compounds were selected and tested in the enzymatic assays. Among them, seven compounds with the most promising MTase inhibitory activities were tested in cellular assays for their antiviral activities, displaying low micromolar potencies and high selectivity indexes (defined as the ratio between CC_50_ and IC_50_). The best result is represented by hit compound **17**, which has an IC_50_ of 0.5 µM with a selectivity index major of 200.

ADME properties were evaluated, highlighting optimal stability after incubation with microsomes and in plasma. The low toxicity of the series was also confirmed by testing compound **17** in vivo using the *Tenebrio molitor* coleoptera preclinical model.

Even if additional computational studies and co-crystallization with MTase are necessary to elucidate the real binding mode of the series, taken together, our results pave the way for further investigation on the series.

## Figures and Tables

**Table 1:** Chemical structures and enzymatic activity of compounds **1**−**20** selected from commercial databases within the MTase-binding site of ZIKV NS5 along with calculated ADME properties.

Cpd ID	Chemical Structure	IC_50_ (μM) ^a^	Commercial Code	LogS ^b^	LogPo/w
**1**	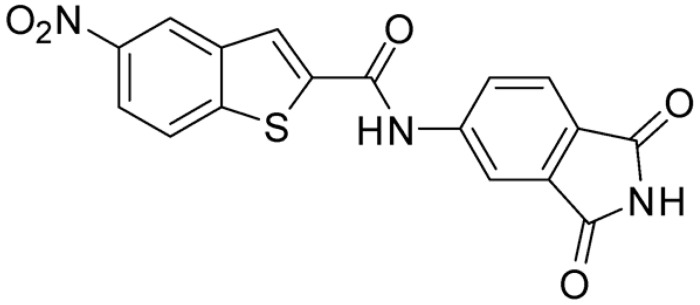	53	FF1105-0186	−4.0	1.20
**2**	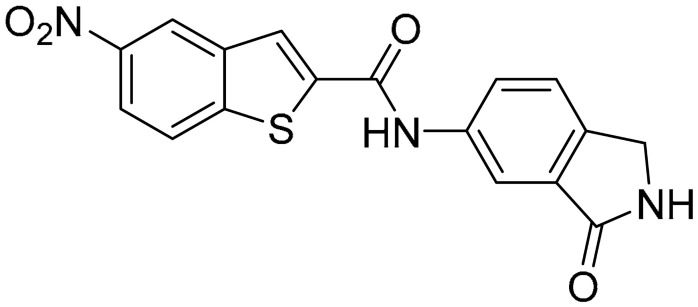	20	Z941456652	−3.97	1.58
**3**	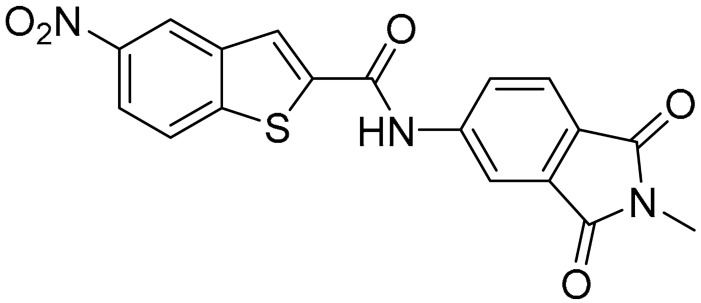	16.55	Z51809662	−4.19	1.85
**4**	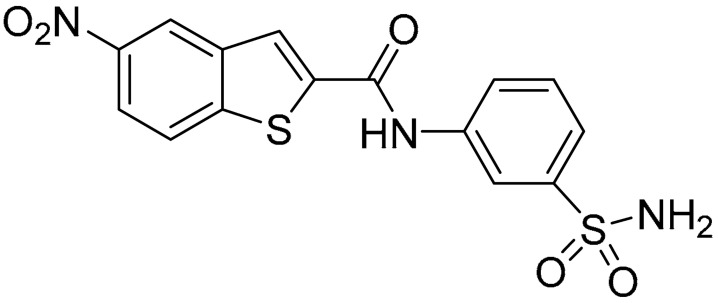	34	Z144391808	−3.87	1.31
**5**	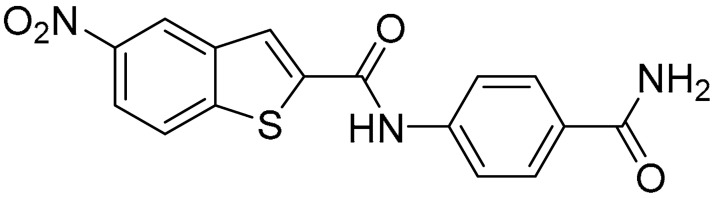	37	Z51981223	−3.86	1.53
**6**	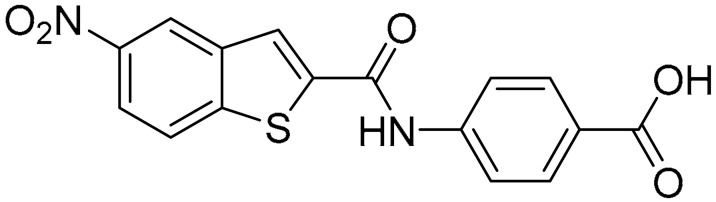	50	Z85880689	−4.44	1.91
**7**	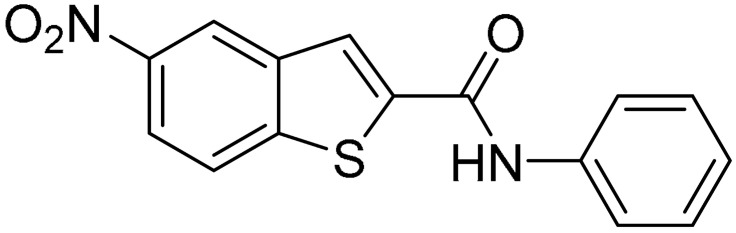	57	Z51820439	−4.44	1.91
**8**	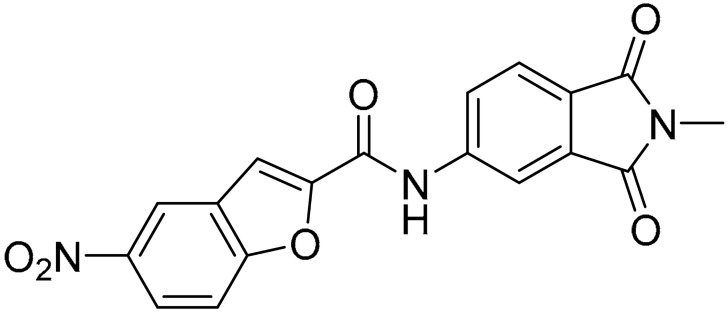	22.57	Z442328966	−3.70	1.68
**9**	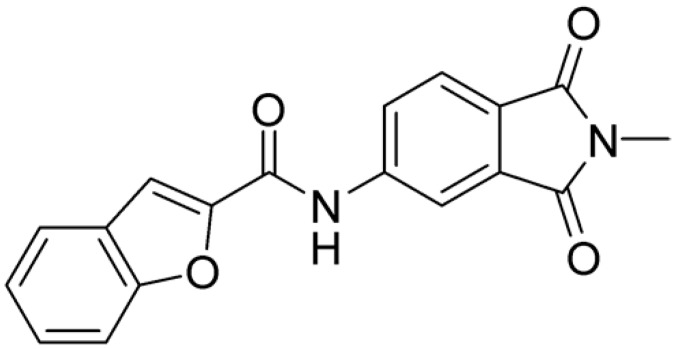	>100	Z27626940	−3.65	2.13
**10**	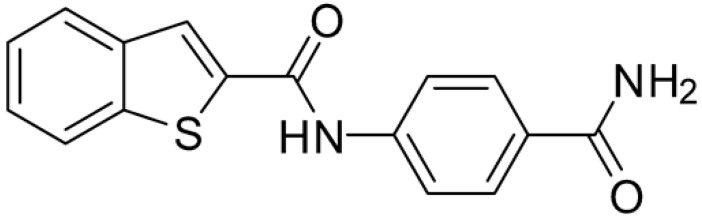	36	Z87538649	−3.82	1.98
**11**	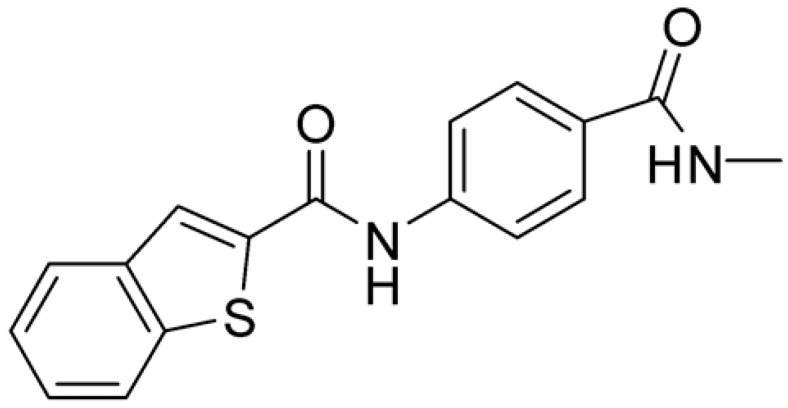	>100	Z87664179	−4.07	2.69
**12**	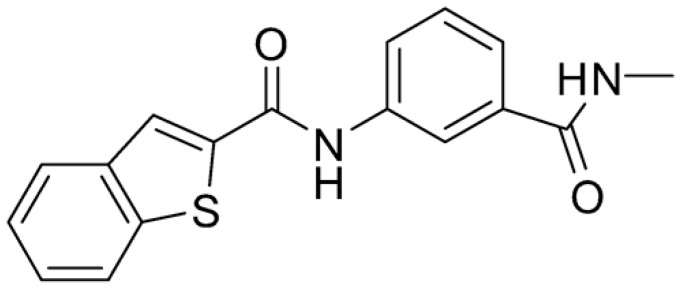	>100	Z88213247	−4.07	2.83
**13**	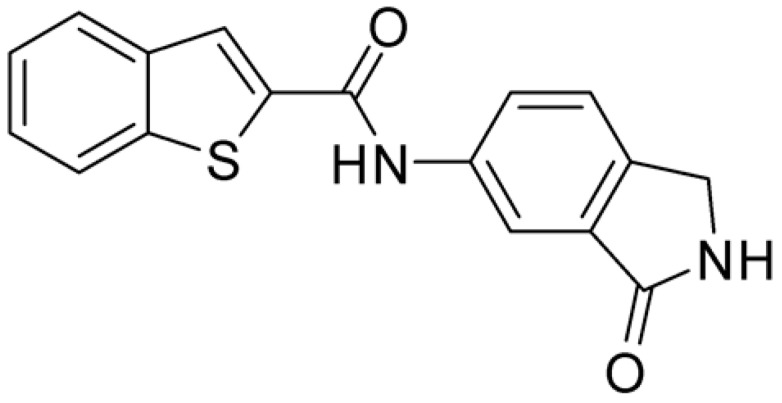	46	Z941455854	−3.92	2.28
**14**	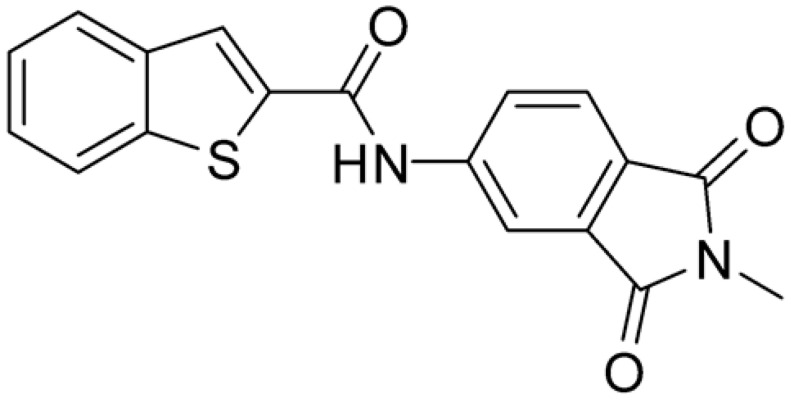	31	Z87640234	−3.87	1.31
**15**	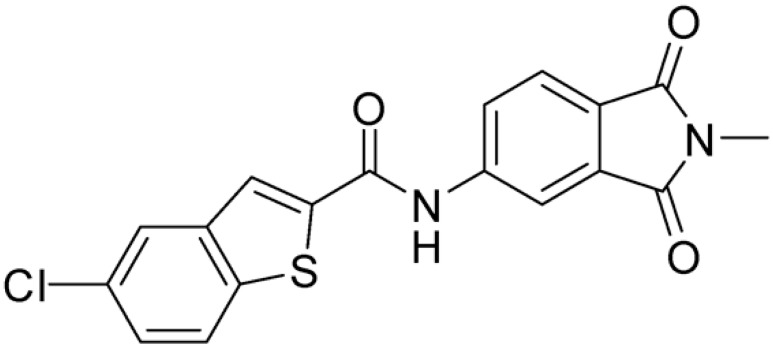	14.15	PV-001792212538	−4.72	2.62
**16**	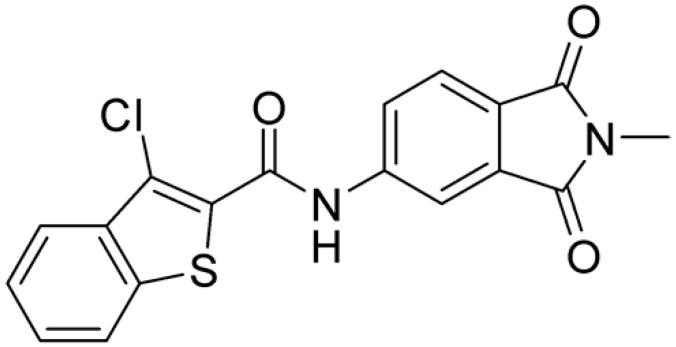	13.14	Z27627244	−4.72	2.78
**17**	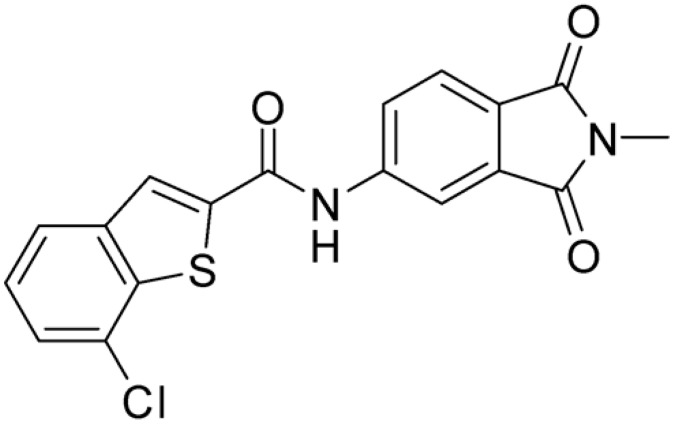	11.37	PV-001792212186	−4.72	2.80
**18**	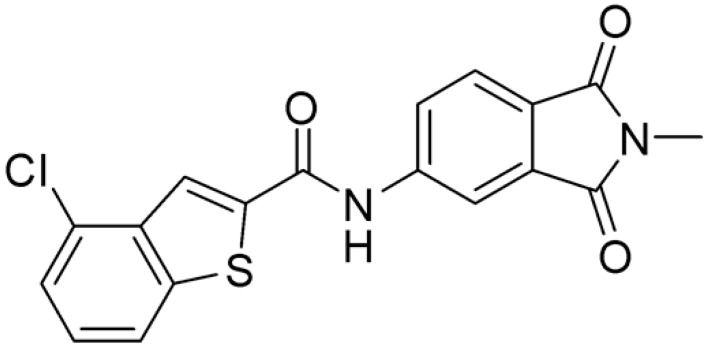	100	Z27626581	−4.72	2.74
**19**	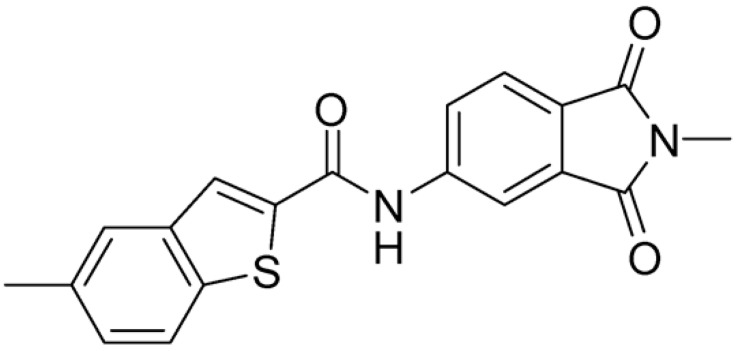	39.98	Z2188823028	−4.43	2.60
**20**	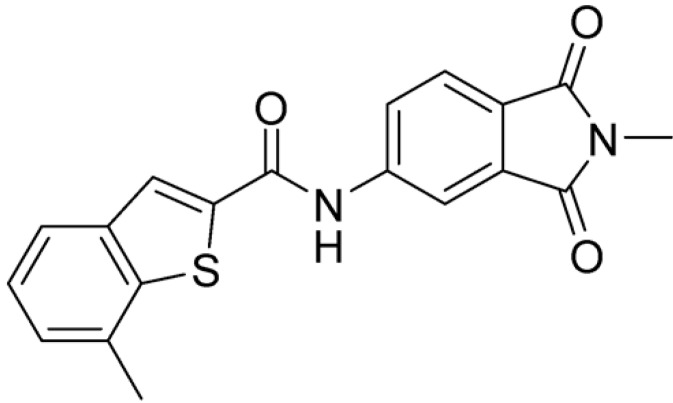	18.93	Z2142703447	−4.43	2.82

^a^ IC_50_: half-maximal inhibitory enzymatic concentration calculated from at least two experiments; ^b^ Aqueous solubility expressed as Log of the solubility.

**Table 2:** Zika anti-NS5 compounds’ inhibitory activity.

Cpd ID	IC_50_ ± SD (μM) ^a^	CC_50_^c^ ± SD (μM) ^b^	SI ^c^
1	>100	>100	-
3	28.6 ± 1.3	>100	>3.49
8	1.9 ± 0.3	>100	>52.63
15	1.4 ± 0.3	>100	>71.43
16	2.6 ± 0.2	>100	>38.46
17	0.5 ± 0.1	>100	>200
20	6.6 ± 0.5	>100	>15.15
ribavirin	95.50 ± 0.6	100 ± 14	1.05

^a^ IC_50_: half-maximal inhibitory concentration calculated in VERO E6 cells from at least two experiments; ^b^ CC_50_: half-maximal cytotoxic concentration evaluated on VERO E6 cells; ^c^ SI: Selectivity Index, calculated as the ratio between CC_50_ and IC_50_.

**Table 3:** Stability studies ^a^.

Cpd ID	HLM Stab.% ± SD ^b^ (M1%, M2%)	Plasma Stab. ^c^ (T_1/2_, h ± SD)
**8**	96.93 ± 0.57 (1.34 ± 0.02, 1.74 ± 0.15)	7.31 ± 0.17
**15**	98.57 ± 0.07 (1.12 ± 0.05, 0.31 ± 0.02)	>24
**17**	96.39 ± 0.11 (1.96 ± 0.07, 1.65 ± 0.08)	>24
**20**	98.40 ± 0.12 (0.83 ± 0.09, 0.77 ± 0.08)	>24

^a^ Values represent the mean values of three independent experiments. ^b^ Human Liver Microsomal Metabolic Stability; M1 and M2 represent the metabolites identified by HPLC-UV-MS analysis. ^c^ Plasma stability is calculated by incubating compounds with human plasma; half-life (T_1/2_) is expressed in hours.

## Data Availability

Compounds are available on request, all data are reported in the manuscript and on Supplementary Materials.

## Supplementary Materials

The following supporting information can be downloaded at https://www.mdpi.com/article/10.3390/ijms25042437/s1.

## References

[1] Woolhouse M., Scott F., Hudson Z., Howey R., Chase-Topping M. (2012). Human Viruses: Discovery and Emergence. Philos. Trans. R. Soc. B Biol. Sci..

[2] Zika Virus Disease. https://www.who.int/health-topics/zika-virus-disease#tab=tab_1.

[3] Orphanet: Malattia del Virus Zika. https://www.orpha.net/consor/cgi-bin/Disease_Search.php?lng=IT&data_id=23562&Disease_Disease_Search_diseaseGroup=zika&Disease_Disease_Search_diseaseType=Pat&Malattia(e)/%20gruppo%20di%20malattie=Malattia-del-virus-Zika%title=Malattia%20del%20virus%20Zika&search=Disease_Search_Simple.

[4] Han Y., Mesplède T. (2018). Investigational Drugs for the Treatment of Zika Virus Infection: A Preclinical and Clinical Update. Expert Opin. Investig. Drugs.

[5] Barrows N.J., Campos R.K., Powell S.T., Prasanth K.R., Schott-Lerner G., Soto-Acosta R., Galarza-Muñoz G., McGrath E.L., Urrabaz-Garza R., Gao J. (2016). A Screen of FDA-Approved Drugs for Inhibitors of Zika Virus Infection. Cell Host Microbe.

[6] Han Y., Pham H.T., Xu H., Quan Y., Mesplède T. (2019). Antimalarial Drugs and Their Metabolites Are Potent Zika Virus Inhibitors. J. Med. Virol..

[7] Touret F., de Lamballerie X. (2020). Of chloroquine and COVID-19. Antivir. Res..

[8] Gonzalez S., Brzuska G., Ouarti A., Gallier F., Solarte C., Ferry A., Uziel J., Krol E., Lubin-Germain N. (2022). Anti-HCV and Zika Activities of Ribavirin C-Nucleosides Analogues. Bioorg. Med. Chem..

[9] Albulescu I.C., Kovacikova K., Tas A., Snijder E.J., van Hemert M.J. (2017). Suramin inhibits Zika virus replication by interfering with virus attachment and release of infectious particles. Antivir. Res..

[10] Sacramento C.Q., de Melo G.R., de Freitas C.S., Rocha N., Hoelz L.V.B., Miranda M., Fintelman-Rodrigues N., Marttorelli A., Ferreira A.C., Barbosa-Lima G. (2017). The clinically approved antiviral drug sofosbuvir inhibits Zika virus replication. Sci. Rep..

[11] Zhou Y., Ray D., Zhao Y., Dong H., Ren S., Li Z., Guo Y., Bernard K.A., Shi P.-Y., Li H. (2007). Structure and Function of Flavivirus NS5 Methyltransferase. J. Virol..

[12] Coutard B., Barral K., Lichière J., Selisko B., Martin B., Aouadi W., Lombardia M.O., Debart F., Vasseur J.-J., Guillemot J.C. (2017). Zika Virus Methyltransferase: Structure and Functions for Drug Design Perspectives. J. Virol..

[13] Tsukamoto Y., Igarashi M., Kato H. (2023). Targeting cap1 RNA methyltransferases as an antiviral strategy. Cell Chem. Biol..

[14] Decroly E., Canard B. (2017). Biochemical principles and inhibitors to interfere with viral capping pathways. Curr. Opin. Virol..

[15] Decombe A., El Kazzi P., Decroly E. (2023). Interplay of RNA 2′-O-methylations with viral replication. Curr. Opin. Virol..

[16] Devarkar S.C., Wang C., Miller M.T., Ramanathan A., Jiang F., Khan A.G., Patel S.S., Marcotrigiano J. (2016). Structural basis for m7G recognition and 2′-O-methyl discrimination in capped RNAs by the innate immune receptor RIG-I. Proc. Natl. Acad. Sci. USA.

[17] Bastiaenen R., Behr E.R. (2011). Sudden death and ion channel disease: Pathophysiology and implications for management. Heart.

[18] Dong H., Chang D.C., Xie X., Toh Y.X., Chung K.Y., Zou G., Lescar J., Lim S.P., Shi P.Y. (2010). Biochemical and Genetic Characterization of Dengue Virus Methyltransferase. Virology.

[19] Iovine N.M., Lednicky J., Cherabuddi K., Crooke H., White S.K., Loeb J.C., Cella E., Ciccozzi M., Salemi M., Morris J.G. (2017). Coinfection with Zika and Dengue-2 Viruses in a Traveler Returning From Haiti, 2016: Clinical Presentation and Genetic Analysis. Clin. Infect. Dis..

[20] Dupont-Rouzeyrol M., O’Connor O., Calvez E., Daures M., John M., Grangeon J.P., Gourinat A.C. (2015). Co-Infection with Zika and Dengue Viruses in 2 Patients, New Caledonia, 2014. Emerg. Infect. Dis..

[21] Zambrano H., Waggoner J.J., Almeida C., Rivera L., Benjamin J.Q., Pinsky B.A. (2016). Zika Virus and Chikungunya Virus CoInfections: A Series of Three Cases from a Single Center in Ecuador. Am. J. Trop. Med. Hyg..

[22] Ramharack P., Soliman M.E.S. (2018). Zika virus NS5 protein potential inhibitors: An enhanced in silico approach in drug discovery. J. Biomol. Struct. Dyn..

[23] Santos F.R.S., Lima W.G., Maia E.H.B., Assis L.C., Davyt D., Taranto A.G., Ferreira J.M.S. (2020). Identification of a Potential Zika Virus Inhibitor Targeting NS5 Methyltransferase Using Virtual Screening and Molecular Dynamics Simulations. J. Chem. Inf. Model..

[24] Bharadwaj S., Rao A.K., Dwivedi V.D., Mishra S.K., Yadava U. (2021). Structure-based screening and validation of bioactive compounds as Zika virus methyltransferase (MTase) inhibitors through first-principle density functional theory, classical molecular simulation and QM/MM affinity estimation. J. Biomol. Struct. Dyn..

[25] Case D.A., Betz R.M., Cerutti D.S., Cheatham T.E., Darden T.A., Duke R.E., Giese T.J., Gohlke H., Goetz A.W., Homeyer N. (2016). Amber 2016.

[26] Miller B.R., McGee T.D., Swails J.M., Homeyer N., Gohlke H., Roitberg A.E. (2012). MMPBSA.Py: An Efficient Program for End-State Free Energy Calculations. J. Chem. Theory Comput..

[27] Humphrey W., Dalke A., Schulten K. (1996). VMD: Visual Molecular Dynamics. J. Mol. Graph..

[28] Schrödinger (2017). 2017-2: LigPrep.

[29] Schrödinger (2017). 2017-2: Glide.

[30] Friesner R.A., Murphy R.B., Repasky M.P., Frye L.L., Greenwood J.R., Halgren T.A., Sanschagrin P.C., Mainz D.T. (2006). Extra Precision Glide: Docking and Scoring Incorporating a Model of Hydrophobic Enclosure for Protein-Ligand Complexes. J. Med. Chem..

[31] Daina A., Michielin O., Zoete V. (2017). SwissADME: A Free Web Tool to Evaluate Pharmacokinetics, Drug-Likeness and Medicinal Chemistry Friendliness of Small Molecules. Sci. Rep..

[32] Kushwaha N., Kaushik D. (2016). Recent Advances and Future Prospects of Phthalimide Derivatives. J. Appl. Pharm. Sci..

[33] Brai A., Poggialini F., Vagaggini C., Pasqualini C., Simoni S., Francardi V., Dreassi E. (2023). Tenebrio molitor as a Simple and Cheap Preclinical Pharmacokinetic and Toxicity Model. Int. J. Mol. Sci..

[34] Wolber G., Langer T. (2005). LigandScout: 3-D Pharmacophores Derived from Protein-Bound Ligands and Their Use as Virtual Screening Filters. J. Chem. Inf. Model..

[35] Peyrane F., Selisko B., Decroly E., Vasseur J.J., Benarroch D., Canard B., Alvarez K. (2007). High-yield production of short GpppA- and ^7Me^GpppA-capped RNAs and HPLC-monitoring of methyltransfer reactions at the guanine-N7 and adenosine-2′O positions. Nucleic Acids Res..

[36] Rango E., D’Antona L., Iovenitti G., Brai A., Mancini A., Zamperini C., Trivisani C.I., Marianelli S., Fallacara A.L., Molinari A. (2021). Si113-prodrugs selectively activated by plasmin against hepatocellular and ovarian carcinoma. Eur. J. Med. Chem..

