# Supplementary material for: Identification of Novel Non-Nucleoside Inhibitors of Zika Virus NS5 Protein Targeting MTase Activity
Source: Int J Mol Sci. 2024 Feb 19;25(4):2437. doi: 10.3390/ijms25042437 (PMC10888717; doi:10.3390/ijms25042437)
Supplement: Supplementary file 1 [file ijms-25-02437-s001.zip › ijms-2862282-supplementary.pdf]

International Journal of
Molecular Sciences

MDPI

Article

Identification of Novel Non-Nucleoside Inhibitors of Zika Virus NS5 Protein Targeting MTase Activity

Diego Fiorucci [1,2†], Micaela Meaccini [1†], Giulio Poli [1,3], Maria Alfreda Stincarelli [4], Chiara Vagaggini [1], Simone Giannecchini [4], Priscila Sutto-Ortiz [5], Bruno Canard [5], Etienne Decroly [5], Elena Dreassi [1] and Annalaura Brai [1*] and Maurizio Botta [1]

[1] Department of Biotechnology, Chemistry and Pharmacy, University of Siena, via Aldo Moro 2, 53100 Siena, Italy.
[2] Current Affiliation: Aptuit, an Evotec Company, 37135 Verona, Italy.
[3] Current Affiliation: Department of Pharmacy, University of Pisa, 56126 Pisa, Italy
[4] Department of Experimental and Clinical Medicine, University of Florence, Viale Morgagni 48, 50134, Florence, Italy.
[5] AFMB, Aix-Marseille University, CNRS, UMR 7257, Case 925, 163 Avenue de Luminy, 13288 Marseille Cedex 09, France.
* Correspondence: annalaura.brai@unisi.it
† The authors equally contribute to the work.

Table of Contents

Figure S1 3
Figure S2 4
Figure S3 4
Table S1 5

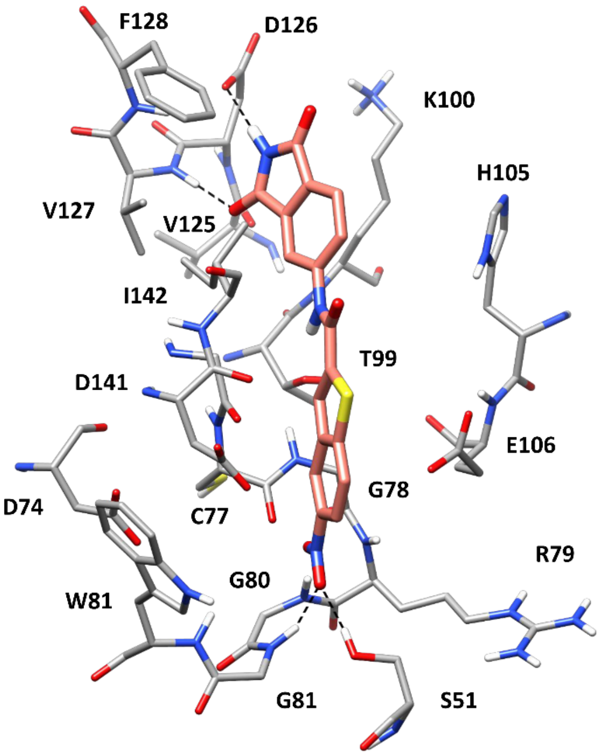

Figure S1. Binding mode of ligand 1 predicted by docking. The hydrogen bonds corresponding to the required pharmacophore features are shown as black dashed lines.

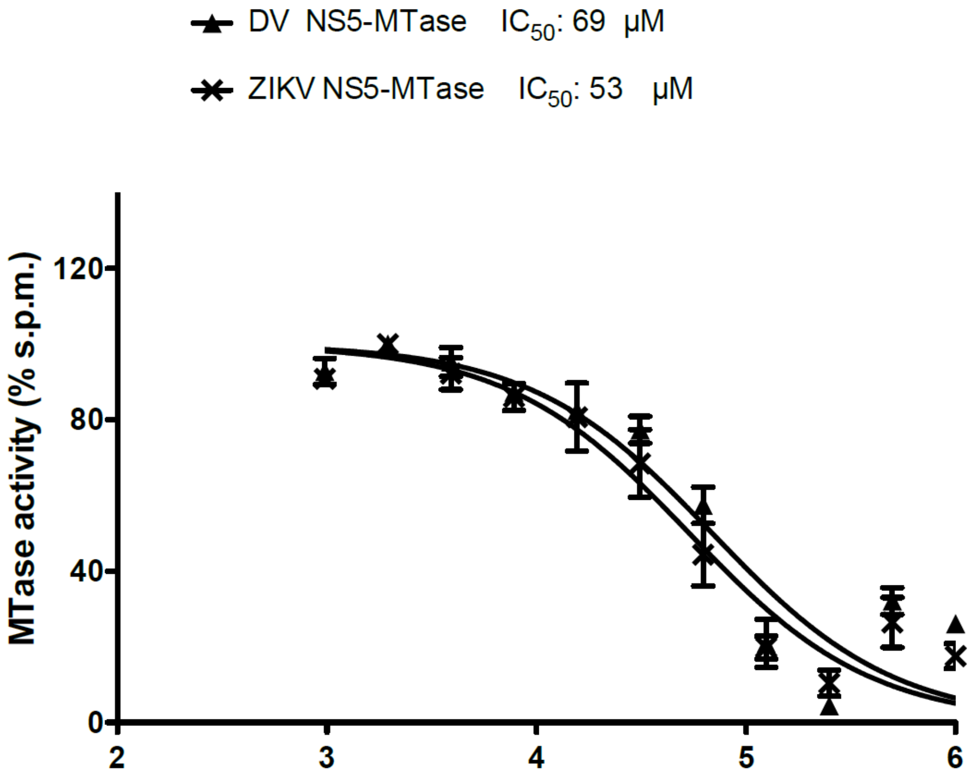

Figure S2. Inhibitory curves for compound **1** vs Dengue (DV) and Zika (ZIKV) virus methyltransferase (Mtase) and IC50 values.

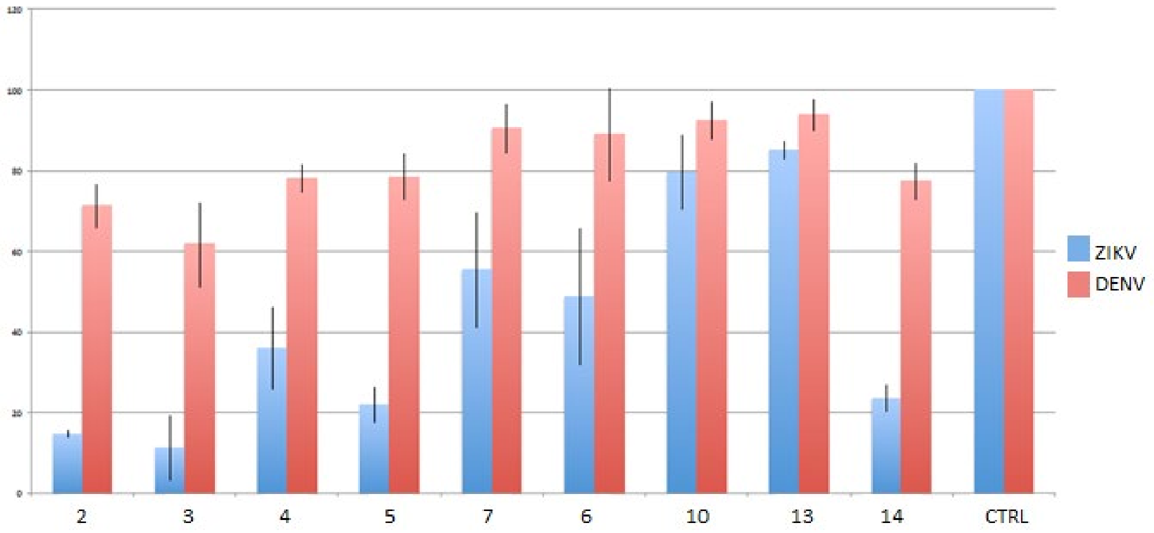

Figure S3. Screening of compounds against ZIKV and DENV N-terminal methyltransferase (Mtase). Only representative compounds are shown.

Table S1. Molecular mechanics-generalized Born surface area (MM-GBSA) values calculated for compounds **1**–**20**.

Compound ID	MMGBSA (kcal/mol)	StDev
**1**	-32.0	4.3
**2**	-33.9	5.1
**3**	-36.2	4.9
**4**	-21.7	3.9
**5**	-23.4	4.5
**6**	-29.8	4.0
**7**	-18.2	4.5
**8**	-28.3	3.9
**9**	-17.8	4.1
**10**	-30.7	3.4
**11**	-22.3	3.7
**12**	-21.6	3.6
**13**	-29.4	4.8
**14**	-26.2	3.1
**15**	-26.4	3.9
**16**	-26.7	4.0
**17**	-28.6	4.1
**18**	-25.2	4.4
**19**	-28.3	3.7
**20**	-28.8	3.2
